# FRET-Based Screening Identifies p38 MAPK and PKC Inhibition as Targets for Prevention of Seeded α-Synuclein Aggregation

**DOI:** 10.1007/s13311-021-01070-1

**Published:** 2021-07-13

**Authors:** Alexander Svanbergsson, Fredrik Ek, Isak Martinsson, Jordi Rodo, Di Liu, Edoardo Brandi, Caroline Haikal, Laura Torres-Garcia, Wen Li, Gunnar Gouras, Roger Olsson, Tomas Björklund, Jia-Yi Li

**Affiliations:** 1 Department of Experimental Medicine, Neural Plasticity and Repair, Lund University, Lund, Sweden; 2 Chemical Biology & Therapeutics, Department of Experimental Medicinal Science, Lund University, Lund, Sweden; 3 Experimental Dementia Research, Department of Experimental Medicine, Lund University, Lund, Sweden; 4 Molecular Neuromodulation, Department of Experimental Medicine, Lund University, Lund, Sweden; 5 Institute of Health Sciences, China Medical University, Shenyang, 110112 China; 6 Lund University, Lund, Sweden

**Keywords:** α-Synuclein, High-throughput screening, Kinase, FRET, Proteomics, Endosome, Lysosome

## Abstract

Aggregation of α-synuclein is associated with neurodegeneration and a hallmark pathology in synucleinopathies. These aggregates are thought to function as prion-like particles where the conformation of misfolded α-synuclein determines the traits of the induced pathology, similar to prion diseases. Still, little is known about the molecular targets facilitating the conformation-specific biological effects, but their identification could form the basis for new therapeutic interventions. High-throughput screening of annotated compound libraries could facilitate mechanistic investigation by identifying targets with impact on α-synuclein aggregation. To this end, we developed a FRET-based cellular reporter in HEK293T cells, with sensitivity down to 6.5 nM α-synuclein seeds. Using this model system, we identified GF109203X, SB202190, and SB203580 as inhibitors capable of preventing induction of α-synuclein aggregation via inhibition of p38 MAPK and PKC, respectively. We further investigated the mechanisms underlying the protective effects and found alterations in the endo-lysosomal system to be likely candidates of the protection. We found the changes did not stem from a reduction in uptake but rather alteration of lysosomal abundance and degradative capacity. Our findings highlight the value high-throughput screening brings to the mechanistic investigation of α-synuclein aggregation while simultaneously identifying novel therapeutic compounds.

**Supplementary Information:**

The online version contains supplementary material available at 10.1007/s13311-021-01070-1.

## Introduction

Alpha-synuclein (α-syn) aggregation is a common feature of synucleinopathies, a group of neurodegenerative diseases including Parkinson’s disease (PD), multiple system atrophy (MSA), and dementia with Lewy bodies (DLB). The deposition of α-syn aggregates presents distinctly depending on the disease. PD and DLB are both characterized by aggregates called Lewy bodies (LB) and Lewy neurites (LN) found in neurons [1]. MSA presents instead with predominantly glial cytoplasmic inclusions (GCI) in oligodendrocytes, but also a degree of neuronal cytoplasmic and intranuclear aggregates [2].

Current hypotheses suggest synucleinopathies are driven by a cell-to-cell spread of misfolded α-syn in a prion-like manner. This mechanism is supported by a progressive temporal spread of synuclein pathology to interconnected brain areas following injection of fibrillar α-syn seeds into the striatum or olfactory bulb [1, 3]. Pathology may also originate in the gastrointestinal tract following injection of recombinant fibrils or patient-derived aggregates and progress toward the brain, in line with Braak’s hypothesis [4, 5]. The spread of pathology has also been reported in patients receiving grafts of fetal mesencephalic dopaminergic neurons, where Lewy pathology was observed during post-mortem autopsy 11–24 years following transplantation [6–9].

A prion-like spread may also account for the heterogeneity of synucleinopathies, as conformational differences may result in altered interactions. Like prion diseases, α-syn has been shown to take on various conformations upon misfolding, which in turn could alter the disease etiology similar to bona fide prion diseases. Such structurally distinct fibrils have been produced in cell-free conditions and were shown to possess a structure-dependent spread of pathology and detrimental effects [10]. Differences in biological activity are also observed for aggregated α-syn isolated from PD and MSA patient brains, where MSA-derived aggregates display a higher seeding efficacy [11]. These conformational and function differences of such pathological aggregates may form as a consequence of the local environment of the affected cell types, as recently reported [12].

While our understanding of the mechanisms involved in α-syn-related pathology has vastly expanded over the last decade, this has not yet translated into treatment options for any synucleinopathy. Immunotherapy [13], gene therapy [14], or anti-sense oligonucleotides [15] are exciting treatment options for neurodegenerative diseases currently under development. Alternative approaches such as small molecule compound screening have also helped identify novel targets or treatments [16]. Annotated libraries with thousands of compounds have been established and are commercially available for the application for appropriate disease models.

To provide a high-throughput screening (HTS) platform, we have leveraged a system based on fluorescence resonance energy transfer (FRET)-based detection of α-syn aggregation by flow cytometry. After validation and characterization of our method, we screened a library of small molecule kinase inhibitors. We identified three novel compounds with strong inhibitory effects on seeded α-syn aggregation in vitro by targeting p38 mitogen-activated protein kinase (p38 MAPK) and protein kinase C (PKC). We further examine the cellular alterations induced by the inhibitors and observe lysosomal-related changes, which are likely the source of the inhibitors’ protective effects.

## Methods

### α-Syn Production

Recombinant α-syn was produced as previously described [17]. Briefly, codon optimized human α-syn was expressed in *E. coli* using a Pet3a inducible plasmid. Bacterial cultures were maintained at 37 °C with 125 rpm shaking until an OD_600_ value between 0.6 and 1.00 was obtained, at which point α-syn expression was induced by 0.4 mM IPTG for 4 h. Cultures were harvested by centrifugation and α-syn purified by heat treatment ion exchange chromatography. Finally, to ensure monomeric α-syn was produced, the isolated protein was run on a size exclusion column (Superdex 75, GE Healthcare) with a Tris buffer (10 mM Tris, 150 mM, NaCl pH 7.5). From the resulting fractions, the central fraction of the monomer peak was collected. Yield was determined by absorbance at 280 nm (Nanodrop, Thermo Fisher) and calculated using an extinction coefficient ε = 5800.1/M/cm.

### Fibril Production

Freshly prepared human recombinant α-syn was adjusted to a concentration of 0.5 µg/µl with Tris buffer (10 mM Tris, 150 mM, NaCl pH 7.5) and incubated for 14 days at 37 °C with 1000 rpm agitation using 3 mm magnets in low binding 1.5 ml Eppendorf tubes. Fibrillation of the monomeric α-syn was confirmed with Thioflavin T readings and transmission election microscopy.

For all experiments using PFFs, cup-horn sonication was performed immediately prior to addition using a Qsonica Q125 Sonicator (Qsonica, Newtown, CT) for 3 min in cycles of 1 s on, 1 s off at 70% amplitude.

### Cloning

Using constructs previously generated in our laboratory carrying α-syn^A53T^-CFP and α-syn^A53T^-YFP as donor plasmids, fluorescently tagged α-syn was PCR amplified and overhangs were added for XbaI/XhoI digestion. By enzymatic digestion and gel purification of the PCR product, the amplicon was inserted into a linearized pHsCXW backbone with the same overhangs. The resulting plasmids (pHsCXW: α-syn^A53T^-CFP and pHsCXW: α-syn^A53T^-YFP) gave a strong uniform CMV-driven expression α-syn carrying the fluorophores of interest.

### Lentivirus Production

Lentivirus production was carried out in HEK293-T cells, using packaging plasmids pMD2G, pMDL, and pRsvREV, and the lentiviral transfer plasmid carrying the expression cassette of interest. Cells were seeded in a Nunc T175 flask at a density of 14·10^6^ cells/flask and incubated overnight. The following day, 2 h before transfection, the culture media was replaced for 16.2 ml fresh media. For lentivirus assembly, 5.1 µg pMD2G, 7.1 µg pMDL, 4.0 µg pRsvREV, and 18.0 µg transfer plasmid was mixed in 1 ml of DMEM without supplements. PEI (102.6 µl) was added to the reaction mix, and the total volume was adjusted to 1.8 ml before vortexing thoroughly. The transfection mix was incubated for 15 min at room temperature before gently adding to the cells. Media was harvested at 48 and 72 h and replaced by new media. After the second harvest, collected media was spun at 800* g*, 4 °C for 10 min to pellet cell debris and filtered through a 0.22 µm filter. The filtrate was transferred to a Beckman Coulter ultracentrifuge tube and spun at 25,000 rpm in SW-32 swing-buckets to concentrate the virus particles. The supernatant was discarded, and the tubes were air dried before adding 80 µl sterile PBS to each tube to resuspend the pellet overnight. After resuspension, the virus suspension was aliquoted and tittered.

### Cell Culture

All cells were cultured at 37 °C, 95% relative humidity, and 5% CO_2_ in DMEM supplemented with 10% FBS and 1% penicillin/streptomycin. Maintenance of the cultures was done by sub-culturing at 80–90% confluence by gentile detachment of the cells, transferring to new culture flasks with fresh media. Ninety-six-well multi-well plates were coated with Collagen G for 1 h and washed twice with PBS before seeding cells. All experiments for direct addition of α-syn fibrils were seeded with 30,000 cells/cm^2^, and experiments using lipofectamine for fibril delivery were seeded with 90,000 cells/cm^2^.

### Generation of FRET-Based Cell Model for α-Synuclein Aggregation

Lentiviral transduction with 1 moI of each α-syn^A53T^-CFP and α-syn^A53T^-YFP was used to facilitate stable integration of the FRET reporter molecules in HEK293T cells. After 1 week of maintaining the culture, cells were harvested by trypsin, pelleted, and resuspended in PBS supplemented with 2% FBS before being passed through a 100 µm cell strainer (Falcon). The resulting single cell suspension was stained with Draq7 (1:10,000) for viability assessment and sorted on a FACSAria II as single cells into a 96 well culture plate with 50% fresh media and 50% conditioned media (conditioned media was prepared taking cells from the parental HEK293T culture, spinning at 800 g for 10 min and filtering through a 0.22-µm syringe filter). Sorted cells were expanded and frozen as monoclonal reporter cells.

### Immunocytochemistry

Cells were washed with PBS, fixed with 4% paraformaldehyde and blocked with PBS + 1% BSA + 0.1% Tween for 1 h. Cells were incubated at 4 °C overnight with primary antibody, 1:1000, in blocking buffer. Antibodies include α-syn (SC12767, Santa-Cruz Biotechnology), p-α-syn (AB51253, Abcam), HSP60 (#4870, Cell Signaling), BIP (AB21685, Abcam) and TFEB (#A303-673A, Thermo Fisher). Excess primary antibody was removed by washing three times with PBS and stained by species-specific secondary antibody for 1 h and DAPI (1 µg/ml) at room temperature. Prior to imaging cells were washed three times with PBS. For staining with CongoRed cells were incubated for 15 min with 1 µM CongoRed (Sigma-Aldrich) in 60% PBS/39% ethanol/20 mM NaOH. Cells were rinsed three times in staining buffer without Congo red followed by five times with PBS before imaging.

### Protein Extraction and Immunoblotting

Extraction of detergent-soluble and detergent-insoluble material was performed as previously described [18], and cells were harvested in cold lysis buffer (20 mM Trizma base, 150 mM NaCl, 1 mM EDTA, 0.25% NP-40, 0.25% Triton X-100, pH 7.4) with protease and phosphatase inhibitors. After 20-min incubation on ice, lysate was spun for 20 min at 4 °C, 14,000* g*. Supernatant was stored as detergent soluble fraction, while the pellet was resuspended in lysis buffer with added 5% (wt/vol) SDS and sonicated by cup-horn at 60% amplitude, 3 s on/off cycles for 15 s.

For immunoblotting, even protein amounts were loaded and separated on mini-PROTEAN TGX precast 4–15% Bis–Tris gels (#4,568,085, Bio-Rad, Copenhagen, Denmark). Using a Bio-Rad Trans-Blot semidry transfer system proteins were transferred to a nitrocellulose membrane (#1,704,159, Bio-Rad) and blocked for 1 h (PBS, 0.1% Tween 20, 3% BSA) at room temperature. Primary antibody (α-syn-211, sc-12767, Santa-Cruz Biotechnology, Heidelberg, Germany) staining was performed, 1:1000, in blocking buffer at 4 °C overnight on a shaking table. After three washes with (PBS, 0.1% Tween 20) incubation with secondary species specific HRP-conjugated antibody was performed in blocking buffer for 1 h prior to development on a Bio-Rad ChemiDocMP imaging system.

### Lysotracker Assessment of Acidified Compartments

To assess the extent of acidic compartments within cells, Lysotracker Deep Red (#L12492, Thermo Fisher) was added to culture medium 30 prior to analysis (1:1000). For live-cell imaging Hoechst 33,342 (#62,249, ThermoFisher) was added at a concentration of 0.1 µg/ml together with Lysotracker, while imaging was performed on a Nikon TI microscope equipped with an OKOlab live-cell chamber. Flow cytometric analysis was performed by harvesting cells by trypsin detachment, resuspension into PBS supplemented with 1 µg/ml DAPI for staining dead cells.

### FITC-Dextran Uptake Assay

To follow endo-lysosomal uptake, we added 10 kDa FITC-dextran (#FD10S-100MG, Sigma-Aldrich) at 0.1 mg/ml and incubate at 37 °C. After 1 h of incubation, cells were washed twice with PBS and harvested by trypsin digest and resuspended in PBS. Cells in suspension were analyzed by flow cytometry on a LSRFortessa ex. 488, em. 530, and ex. 610, for assessment of the pH-sensitive and pH-insensitive parts of the FITC spectrum [19].

### Small Molecule Compound Library Screen

An established small molecule kinase inhibitor library (BML-2832, Enzo Life Sciences) was selected for the screen. All compounds included in the library as well as the resulting z-scores can be found in Supplementary Table 1 (online resource 2). All compounds were dissolved in DMSO at a concentration of 10 mM. For each screen, 10,000 cells/well were seeded in a collagen G-coated 96-well plate the day prior to addition of inhibitors and α-syn fibrils. The following day, inhibitors were added by pipetting robot to each well for the final concentration of 2, 4, and 10 µM after addition of α-syn. Half an hour after addition of inhibitors, α-syn fibrils were added and observed for 48 h by live-cell imaging. At endpoint, the cells were harvested with trypsin and fixed in suspension for 20 min on ice. Fixed cells were washed twice in PBS before analysis of normalized FRET intensity (% FRET^+^  × FRET MFI) by flow cytometry (LSRFortessa). Compounds were ranked based on calculated z-score $\frac{x-{\mu }_{sample}}{{\sigma }_{sample}}$ (*x*: observed value, µ: sample mean, *σ*: sample standard deviation). Threshold for hit compounds was set at z-score =  ± 1.5.

### Image Analysis

Image analysis was performed using Python 3.1 with image analysis pipelines made available through GitHub. Co-localization analysis was performed by calculating the PCC for every pixel in images between the two stainings in question (https://github.com/AlexanderSvan/PCC-colocalization-for-images). Aggregate detection was performed by application of Gaussian blur and a white top hat filter, prior to setting a threshold for aggregates. Object detection was performed using the package Sci-kit image, and aggregates were filtered based on aggregate size (https://github.com/AlexanderSvan/Live-cell-aggregate-count). For TFEB nuclear translocation cells were identified and subdivided into cytosol and nucleus based on DAPI staining. Translocation was calculated as the ratio of TFEB intensity between nucleus and cytosol (https://github.com/AlexanderSvan/TFEB-translocation).

### Proteomics MS

#### Samples

Eighteen cell pellets in total: 5 different drug treatments and 1 vehicle control conditions, 3 biological replicates for each.

#### Sample preparation

Unless stated otherwise, all the chemicals and solvents were purchased from Sigma-Aldrich, Steinheim, Germany.

Suspension traps (S-Traps) based digestion protocol was used for the sample processing according to the manufacturer’s instructions [20]. Cell pellets were lysed by using a detergent based lysis buffer (25 mM dithiothreitol (DTT), 5% sodium dodecyl sulfate (SDS) in 50 mM triethylammonium bicarbonate (TEAB, Thermo Fisher Scientific, Rockford, IL), pH = 8.0). The lysates were boiled for 5 min at 95 °C in a thermomixer with the mixing frequency of 500 rpm and then sonicated to denature proteins, shear DNA, and enhance cell disruption by using a water-bath (4 °C) sonicator Bioruptor (Diagenode) at high power for 40 min (sonication cycle: 15 s on, 15 s off). The samples were centrifuged at 16,000* g* for 10 min at room temperature, and the supernatants were transferred to new tubes. The concentration of protein was determined by using Pierce™ 660 nm Protein Assay Reagent with Ionic Detergent Compatibility Reagent (IDCR) (Thermo Fisher Scientific, Rockford, IL). Around 300 µg of cell lysates for each sample was alkylated with 50 mM iodoacetamide (IAA) for 30 min in the dark. Afterward, each sample was acidified by aqueous phosphoric acid (Merck) with the final concentration of 1.2% and then seven volumes of S-Trap binding buffer (90% methanol, 100 mM TEAB, pH = 7.1) was added. After gentle mixing, the protein solution was loaded directly onto the S-Trap spin column (Protifi, Huntington, NY) without any column pre-equilibration, and spun at 4000* g* for 30 s. The S-Trap column was washed 3 times by using 400 µl S-Trap binding buffer. Each sample was digested with 95 µl 50 mM TEAB containing Lys-C (FUJIFILM Wako Chemicals U.S.A. Corporation) at an enzyme/protein ratio of 1:50 w/w for 2 h at 37 °C and further digested with 30 µl 50 mM TEAB containing trypsin (Sequencing Grade Modified, Promega, Madison, WI) at a trypsin/protein ratio of 1:50 w/w overnight at 37 °C. The peptides were eluted by three stepwise buffers with 80 µl of each, including 50 mM TEAB, 0.2% formic acid (FA) in water and 0.2% FA in 50% acetonitrile (ACN). The three-step elutions were pooled together and dried in a SpeedVac (Concentrator plus Eppendorf).

The peptide concentration was determined by using the Pierce Quantitative Colorimetric Peptide Assay (Thermo Fisher Scientific, Rockford, IL) and 180 µg peptides from each sample were loaded onto MacroSpin column (NestGroup, Southborough, MA) for desalting. In brief, the spin column was primed and equilibrated with 80% ACN/0.1% TFA and 0.1% TFA, respectively, and was washed with 0.1% TFA after sample loading. The peptides were eluted with 80% ACN/0.1% TFA, dried in the SpeedVac and stored in a −80 °C freezer until further process.

Phosphor enrichment was performed on the Agilent AssayMAP Bravo Platform (Agilent Technologies, Inc.) and phosphorylated peptides were enriched by using 5 μl Fe(III)-NTA cartridges. Following the Phospho Enrichment v2.0 protocol, the cartridges were primed with 50% ACN/0.1% TFA and equilibrated with 80% ACN/0.1% TFA. Peptides from desalting step were re-suspended with 80% ACN/0.1% TFA and loaded onto the cartridge for the enrichment. The phosphorylated peptides were eluted with 5% ammonia directly into 50% FA after being washed with 80% ACN/0.1% TFA, lyophilized in the SpeedVac and stored in a −80 °C freezer until LC–MS/MS analysis.

### LC MS/MS Analysis

The LC MS/MS analysis was performed on Tribrid mass spectrometer (MS) Orbitrap Fusion equipped with a Nanospray source and coupled with an EASY-nLC 1000 ultrahigh pressure liquid chromatography (UHPLC) pump (Thermo Fischer Scientific, San Jose, CA). One microgram peptides for global proteomics or all the enriched phosphorylated peptides were loaded and concentrated on an Acclaim PepMap 100 C18 precolumn (75 μm × 2 cm, Thermo Scientific, Waltham, MA) and then separated on an Acclaim PepMap RSLC column (75 μm × 25 cm, nanoViper, C18, 2 μm, 100 Å) with the column temperature of 45 °C. Peptides were eluted by a nonlinear 2 h gradient at the flow rate of 300 nL/min from 2% solvent B (0.1% FA/ACN)/98% Solvent A (0.1% FA in water) to 40% solvent B.

The Orbitrap Fusion was operated in the positive data-dependent acquisition (DDA) mode for both global proteomics and phosphor-proteomics. Full MS survey scans from m/z 350–1500 with a resolution 120,000 were performed in the Orbitrap detector. The automatic gain control (AGC) target was set to 4 × 10^5^ with an injection time of 50 ms. The most intense ions (up to 20) with charge state 2–7 from the full MS scan were selected for fragmentation. MS2 precursors were isolated with a quadrupole mass filter set to a width of 1.2 m/z for global proteomics and 0.7 for phosphor-proteomics. Precursors were fragmented by higher energy collision dissociation (HCD) and detected in Orbitrap detector with the resolution of 30,000 for global proteomics and 60,000 for phosphor-proteomics. The normalized collision energy (NCE) in HCD cell was set at 30%. The values for the AGC target and injection time were 5 × 10^4^ and 54 ms for global proteomics and 110 ms for phosphor-proteomics, respectively. The duration of dynamic exclusion was set at 45 s and the mass tolerance window at 10 ppm.

### Data Analysis

All the MS raw files were submitted to Proteome Discoverer 2.3 (Thermo Scientific) for identification and quantification. The search was performed against the Homo sapiens UniProt revised database with the Sequest HT search engine and decoy database containing reversed version of all protein sequences used to monitor false discovery rate (FDR). Carbamidomethylation of cysteine residues was set as fixed modification, and methionine oxidation, protein N-terminal acetylation, and phosphorylation of serine, threonine, and tyrosine were set as variable modifications. For peptide identification, precursor mass tolerance was set at 15 ppm and fragment mass tolerance at 0.05 Da, respectively. A maximum of two missed cleavage sites was allowed. The IMP-ptmRS algorithm was used to score phosphorylation sites with a site probability threshold > 75. After database searching, 1% FDR for both peptide-spectrum match (PSMs) and peptides was applied to filter out the wrong peptides. For statistical analyses, label-free quantification (LFQ) data was log_2_-transformed and normalized to median value of each sample. Multiple-sample test was performed within the Perseus software, with Dunnett’s T3 post hoc test. All raw data is made availeable through the PRIDE database under proteomeXchange ID PXD024508 (Available at: http://www.ebi.ac.uk/pride/archive/projects/PXD024508) 

### Functional Analysis

We performed functional analysis using the web tool DAVID [21, 22] with the lists of differentially expressed genes from analysis of the proteome and phosphor-proteome as input.

### Statistical Analysis

For all multiple comparisons, one-way ANOVA has been applied with a post hoc test depending on the experiment. Multiple comparisons were performed to a singular control, with a Dunnett’s T3 post hoc to correct for multiple comparisons. For experiments where equal variance could not be assumed, the Welch and Brown–Forsythe post hoc was applied instead.

## Results

### Generation and Characterization of a FRET-Based Reporter System for α-Syn Aggregation

**Fig. 1:**
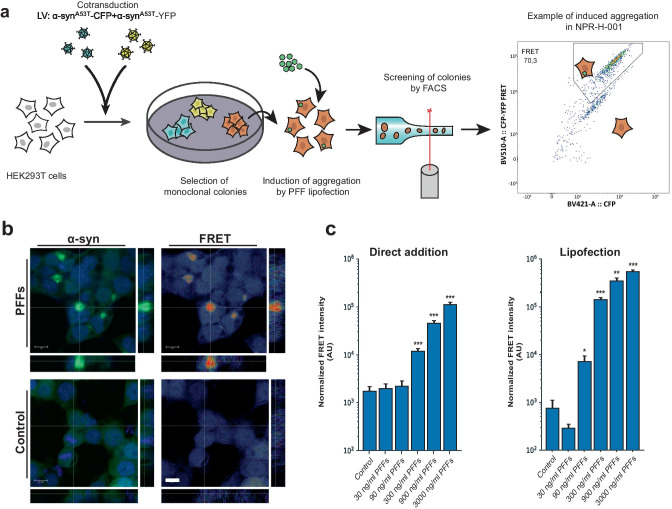
Monoclonal α-syn aggregate FRET reporter cells allow inclusion-specific detection of aggregates. **a** HEK293T cells were simultaneously transduced with lentivirus encoding α-syn^A53T^-CFP and -YFP. Monoclonal cell lines were established by FACS single-cell sorting. Clonal lines were compared for brightness and signal-to-noise ratio of FRET by induction of aggregation by lipofection of α-syn PFFs to the biosensor lines. **b** Confocal microscopy confirmed a strong induction of FRET signal overlapping with the YFP signal, and no induced FRET signatures when treated with monomeric α-syn. cSensitivity assessment by PFF titration and FRET detection by flow cytometry of both direct addition and lipofection with PFFs (direct addition *n* = 6, lipofection *n* = 4). Bar chart values show mean ± SD, *p < 0.05, **p < 0.005, ***p < 0.001. Statistical testing was performed using Brown–Forsythe and Welch one-way ANOVA for multiple comparisons to a control group

To generate a FRET-based α-syn aggregation reporter, we transduced HEK293T cells with lentiviruses encoding α-syn^A53T^-CFP and α-syn^A53T^-YFP. We selected the A53T mutant variant of α-syn, partly due to its relevance as one of the autosomal dominant variants of PD [23], but also due to the increased propensity to aggregate [24, 25]. By fluorescence activated cell sorting (FACS) sorting on the double-positive cell population, we generated 12 monoclonal cell lines. The monoclonal lines, established by single-cell sorting and subsequent clonal expansion, were treated with PFFs and liposomes to facilitate direct delivery of aggregates and induce aggregation (Fig. 1a). The final reporter cell line was selected based on normalized FRET intensity (FRET means fluorescence intensity multiplied by %FRET positive) and signal-to-noise ratio of the FRET signal (data not shown) as detected by flow cytometry. The cell line is from hereon referred to as NPR-H-001.

As flow cytometry detects FRET signal on a per-cell basis, we sought to confirm the observed FRET signal originated from induced aggregates. NPR-H-001 cells were treated with PFFs and liposomes, followed by fixation 24 h after addition for confocal microscopic visualization (Fig. 1b). The detected FRET signal was confirmed to mainly derive from the induced aggregates. We next sought to determine the sensitivity by which induced aggregation could be detected in NPR-H-001 reporter cells. Inducing aggregation with increasing amounts of PFFs delivered either directly or by liposomes, we significantly detect induced aggregation by PFFs by concentrations as low as 300 ng/ml (*p* < 0.0001) and 90 ng/ml (*p* = 0.039), respectively. We further assessed the z-factor of our NPR-H-001 reporter cells, a parameter frequently used to evaluate suitability to HTS, reflecting the separation between the control and maximal induction. Using NPH-H-001 we obtained a z-factor of 0.64 and 0.79 when induced with 3000 ng/ml PFFs, for direct and lipofection respectively, indicating the assay is well suited for screening purposes.

### Induced Aggregates Recapture Hallmarks of Synucleinopathies

In the reporter cell line generation and characterization, we have applied liposomes for the delivery of PFFs. Liposome-mediated delivery facilitates the cytosolic entry of PFFs prior to initiation of aggregation, precluding the cytosolic entry from being studied. Therefore, we sought to assess if the direct addition of PFFs to the media could offer a comparable induction of aggregation, including endocytic events in the experimental paradigm. To this end, we compared the capacity of the two methods of inductions to induce α-syn seeding and deposition of insoluble α-syn.

**Fig. 2:**
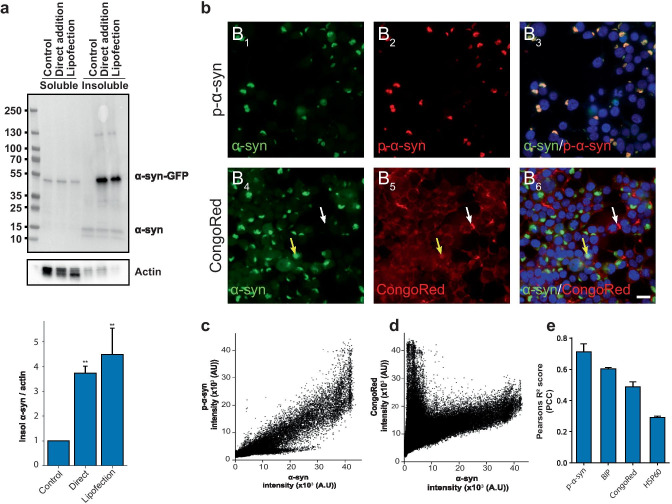
Induction of α-syn aggregation in NPR-H-001 cells recaptures key hallmarks of synucleinopathy. **a** Treatment of Hek293T α-syn^A53T^-GFP with PFFs leads to a significant increase in phosphorylated α-syn compared to treatment with monomeric α-syn. Quantification of Western blot analysis showed levels of phosphorylated α-synuclein increased 3.7 (*p* < 0.009) and 4.4 (*p* < 0.003) folds with direct addition and lipofection, respectively, when compared to control treatment (*n* = 3). **b** α-Syn forms aggregates visible as compacted green inclusions, and colocalizes with phosphorylated α-synuclein as seen in B3. Staining with CongoRed reveals two populations of aggregates, double-positive for GFP and Congo red (B_6_ yellow arrow) and Congo red positive GFP negative (B_6_ white arrow) (scale bar 20 µm). **c**–**d** Correlation scatter plot between α-syn-GFP and p-α-syn **c** and Congo red **d**. **e** PCC (R2) for p-α-syn (0.71 ± 0.05), Congo red (0.48 ± 0.03), and the chaperone proteins BIP (0.60 ± 0.01) and HSP60 (R2 = 0.29 ± 0.008) (*n* = 3). Bar charts show mean ± SD, *p < 0.05, **p < 0.005, ***p < 0.001. Statistical testing was performed using one-way ANOVA with Dunnett’s T3 post hoc test for multiple comparisons to a control group, except **e** where comparisons between groups were not meaningful

As lipofection seeds aggregation more rapidly, cells treated with liposomes and PFFs were harvested 24 h after addition compared to direct addition after 48 h. We then performed sequential extraction of the soluble and insoluble fractions from protein lysates of HEK293T cells with induced α-syn aggregation or control and assessed differences in accumulation of insoluble and phosphorylated α-syn (p-α-syn) via direct addition or lipofection-mediated delivery (Fig. 2a). While the fraction of Triton X-100 soluble p-α-syn remained unaltered, a 3.7-fold and 4.4-fold increase was observed in the insoluble fraction of direct addition and lipid delivery, respectively (Fig. 2a). The formation of insoluble aggregates seen for both delivery methods suggests that both are viable options for studying α-syn aggregation using our novel NPR-H-001 reporter cells. Therefore, we used direct addition for all experiments to induce aggregation to allow uptake and internalization effects to be reflected in our model.

We further characterized the induced aggregates for markers of pathology related to synucleinopathy. First, we assessed the extent to which induced aggregates were phosphorylated by immunostaining with anti-phosphorylated (S129)-α-syn (pS129). We observed a strong positive correlation defined by Pearson’s correlation coefficient (PCC) (*R*^2^ = 0.71 ± 0.05) (Fig. 2b (B_1–3_) and c). Aggregated α-syn is also known to be rich in cross-β-sheet structure as this is the major fold of amyloids. Staining for the amyloid structure with CongoRed resulted in a moderate overall colocalization (*R*^2^ = 0.48 ± 0.03). However, two populations of CongoRed staining were observed, as evident by the correlation scatterplot (Fig. 2b (B_4–6_) and d). One population with intense CongoRed staining and no GFP intensity (Fig. 2 (B_4–6_), white arrow) and the other with moderate CongoRed but high GFP intensity (Fig. 2 (B_4–6_), yellow arrow). These observed populations likely stem from extracellular PFFs which still have not been internalized and intracellular seeded aggregates. Additionally, we also stained for BIP and HSP60 as these are chaperones frequently associated with protein misfolding. We observed a moderate correlation of the induced aggregates with the chaperones BIP (*R*^2^ = 0.60 ± 0.01) and HSP60 (*R*^2^ = 0.29 ± 0.008), respectively (Supplemental Fig. 1, online resource 1). Taken together, the analyses of the induced aggregates indicate that key pathological features are shared with aggregates found in synucleinopathies, proving the relevance of this seeded aggregation model as an experimental tool for studying synucleinopathies.

### High-throughput Screening Identifies Potential Modulators of α-Syn Aggregation

To identify possible modulators of α-syn aggregation and showcase the NPR-H-001 model for HTS, we performed a small molecule compound screen. We focused our initial screen on kinase inhibitors, as kinases have been implicated in various aspects of PD pathology. Mutations in the gene encoding the kinase leucine-rich repeat kinase 2 have been shown to cause an autosomal dominant form of PD, based on its effects on intracellular trafficking [26]. Furthermore, a main marker for synuclein pathology is its phosphorylation on serine 129, which plays a role in the aggregate formation and toxicity [27, 28].

**Fig. 3:**
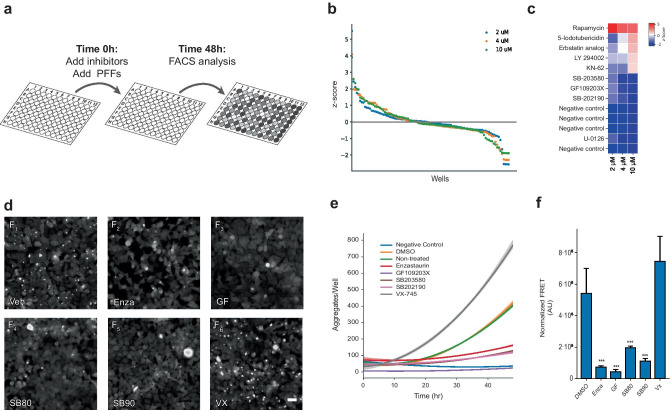
High-throughput screening identifies three kinase inhibitors with potent preventative effects on α-synuclein seeding. **a** Schematic workflow of kinase inhibitor screening using NPR-H001 cells. Assay plates seeded with reporter cells are pre-treated with inhibitors prior to PFF addition and analyzed by flow cytometry after 48 h. **b** Resulting z-scores from kinase inhibitor screening distributes as a waterfall plot with the majority displaying little effect and few tail-end candidates showing large alterations. **c** Ranked representation of samples with z-scores beyond the 1.5 threshold identifies compounds with potential impact on α-synuclein aggregation. **d** Single compound validations show a visual reduction in formed α-syn aggregates 48 h following PFF addition (scale 40 µm) **e**, with differences already being detectable 20 h post PFF addition **e**. **f** Using NPR-H-001 reporter cells to confirm the significant reduction in induced aggregation (Enza: *p* < 0.001, GF: *p* < 0.001, SB80: *p* < 0.001, SB90: *p* < 0.001) following compound addition by flow cytometry. Bar chart shows mean ± SD, ***p < 0.001. Statistical testing was performed using one-way ANOVA with Dunnett’s T3 post hoc test for multiple comparisons to a control group

NPR-H-001 reporter cells were pretreated for 30 min with inhibitors at three concentrations (2, 4, and 10 µM) prior to the addition of PFFs. At the 48-h endpoint following addition of PFFs, cells were fixed and assessed for FRET signal by flow cytometric analysis (Fig. 3a). We calculated the z-factor across all three experimental repetitions of the 10 µM screen, resulting in a z-factor of 0.48 ± 0.1 (mean ± SD), suggesting that the assay is well suited for HTS.

Hit-compounds were selected from cells screened with 10 µM inhibitors with the success criteria defined as a z-score of −1.5. Negative controls, with no induced aggregation, were found to have an average z-score of −1.88 ± 0.01 (mean ± SD), indicating that hit compounds passing the threshold would have a strong preventative effect on induced α-syn aggregation (Supplementary Table 1, online resource 2). Beyond the threshold for all concentrations, we identified 4 inhibitors (SB203580 (SB80), GF109203X (GF), SB202190 (SB90), and U-0126) with strong reduction of normalized FRET signal. Of the four candidate compounds, two were inhibitors of p38 MAPK, one inhibits PKC, and the last targets MEK. We also observed 5 compounds with z-scores surpassing vehicle control (rapamycin, 5-iodotubericidin, Erbstatin analogue, LY29002, and KN-62), indicating an exacerbation of the aggregate phenotype (Fig. 3b–c). This increase in FRET signal was seen to be dose-dependent except for rapamycin which maintained a z-score of 4.49 ± 0.87 (mean ± SD).

To validate the aggregation-inhibition potential of candidate compounds, we assessed the efficacy for each inhibitor by flow cytometry and live-cell microscopy. For comparison, we included two additional inhibitors that have previously been used in clinical testing, though not for in relation to α-syn, with the same targets as the identified compounds, enzastaurin (Enza) targeting PKC (Trial ID: NCT03263026) and VX-745 (VX) targeting p38 MAPK (Trial ID: NCT02423200).

First, we treated NPR-H-001 cells with inhibitors and assessed the aggregation state by flow cytometry, in the same way as was performed for the screen. The compound treatment resulted in a significant decrease in aggregate load for Enza, GF, SB80, and SB90 (Enza 7.4-fold, *p* < 0.001; GF 12.4-fold, *p* < 0.001; SB80 2.7-fold, *p* < 0.001; SB90 4.8-fold, *p* < 0.001). Contrary to the other inhibitors, VX displayed no significant change in aggregate load (Fig. 3d).

We next sought to assess the impact of inhibitor treatment of the kinetics of induced α-syn aggregation. For this, we performed live-cell microscopy with a HEK293T cell line with the single α-syn^A53T^-GFP fusion construct. Similar to the assessment by flow cytometry, a marked reduction in the quantity of α-syn aggregates was observed by live-cell microscopy for all inhibitors tested except VX (Fig. 3e–f). Rather VX led to a significant increase in aggregate load and early detection (~ 10 h following PFF addition) (Fig. 3e). We further examined the effects of the identified inhibitors on WT α-syn-GFP expressing cells, where a significant decrease in aggregate load was observed for Enza, GF, SB80, and SB90 but not VX (Supplemental Fig. 2).

Taken together, the outcome of the kinase inhibitor screen shows the applicability of the NPR-H-001 reporter for HTS, where we identified 3 compounds with strong inhibition of α-syn aggregation by targeting p38 MAPK (SB80 and SB90) and PKC (GF).

### Changes in the Phosphorylation State of Endocytosis-Related Proteins Differentiate PKC from p38MAPK Inhibitors

To investigate the changes induced by the kinase inhibitors, we compared the phospho-proteome of inhibitor-treated cells against vehicle-treated controls as immediate effects of kinase inhibition would result in an altered phosphorylation profile. To this end, we collected lysate from inhibitor-treated cells and enriched part of the lysate for phosphoproteins. The enriched protein sample was analyzed by mass spectroscopy to identify differentially phosphorylated proteins.

**Fig. 4:**
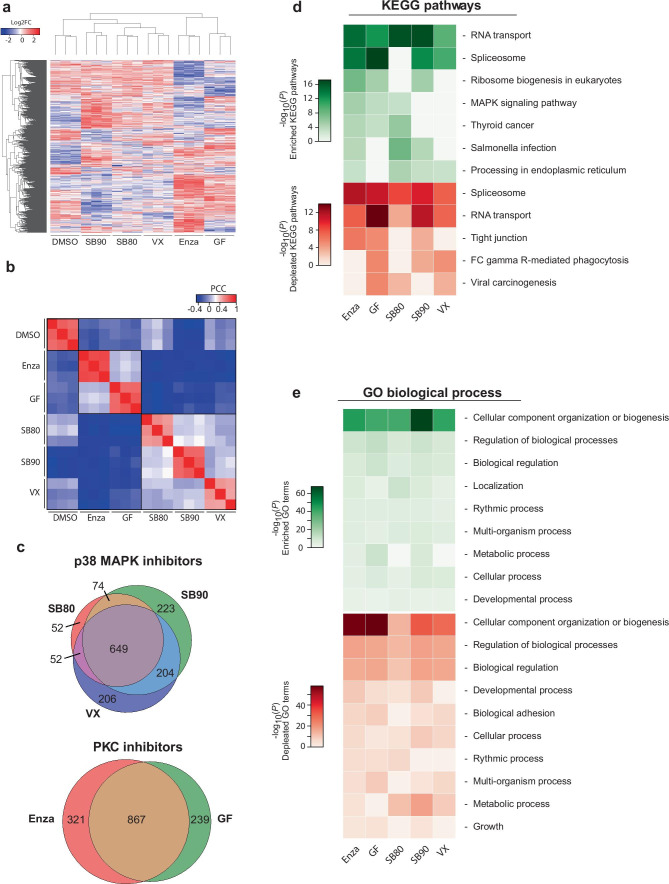
Phospho-proteome shows alterations in protein translation and cellular component organization/biogenesis following inhibitor treatment. **a** Unsupervised clustering of altered phospho-proteome (log2FC) group samples correctly based on target pathways. **b** Reproducibility between replicates and similarity among inhibitors with the same targets give rise to defined clusters based on PCC. **c** Contrasting the altered phospho-proteome depending on inhibitor target shows a high degree of overlap, with 44.5% for p38 MAPK and 60.8% for PKC. **d**, **e** Functional analysis using DAVID of the altered phospho-proteome identified two main processes depending on the analysis employed. **d** KEGG analysis indicated the main alterations, both for increased (green) and decreased (red) phosphorylation being RNA transport and spliceosome. In contrast, GO analysis displayed only one major profile found in both negatively and positively altered, being cellular component organization and biogenesis

We detected 11,620 phospho-epitopes from a total of 4035 proteins, with 3230 epitopes identified as differentially regulated in the inhibitor-treated cells compared to the control. In line with our expectations, clustering of samples based on intensities of detected proteins showed higher similarity between inhibitors with related molecular targets, either PKC or p38 (Fig. 4a). The high PCC of detected protein intensities (*R*^2^ < 0.9) among replicates for each inhibitor supports the reproducibility of the treatment. However, we also observe elevated correlation between inhibitors with the same target indicating similarity in the altered phosphor-proteome (Fig. 4b). The target specificity is also reflected in the overlap of significantly altered phospho-epitopes with a minimum log_2_fold change (log2FC) of 0.5, where 44.5 and 60.8% are shared between the p38 and PKC targeting inhibitors, respectively (Fig. 4c).

Using the altered phospho-proteome, we performed functional analysis to identify altered molecular pathways and processes using KEGG pathway analysis and gene ontology (GO) biological processes, respectively (Fig. 4d, e). From KEGG pathway analysis, the strongest profile altered for all compounds is related to transcription/translation. Both spliceosome and RNA transport were among the most altered, both negatively (Fig. 4d, red) and positively regulated (Fig. 4d, green). Interestingly, the PKC targeting compounds also lead to increased MAPK signalling, as well as for SB80 (Fig. 4d). Analysis of altered biological processes with GO identified cellular organization and biogenesis, as the main process with altered phosphorylation. These results indicate that translation machinery and organelle biogenesis are the major altered pathways in response to the treatments with the inhibitory compounds.

### Proteome Analyses Indicate Alterations in Endo-lysosome Pathways upon PKC and p38 MAPK Inhibition

With protein production and the translation machinery mostly affected within the phospho-proteome, we sought to investigate changes in the global proteome.

**Fig. 5:**
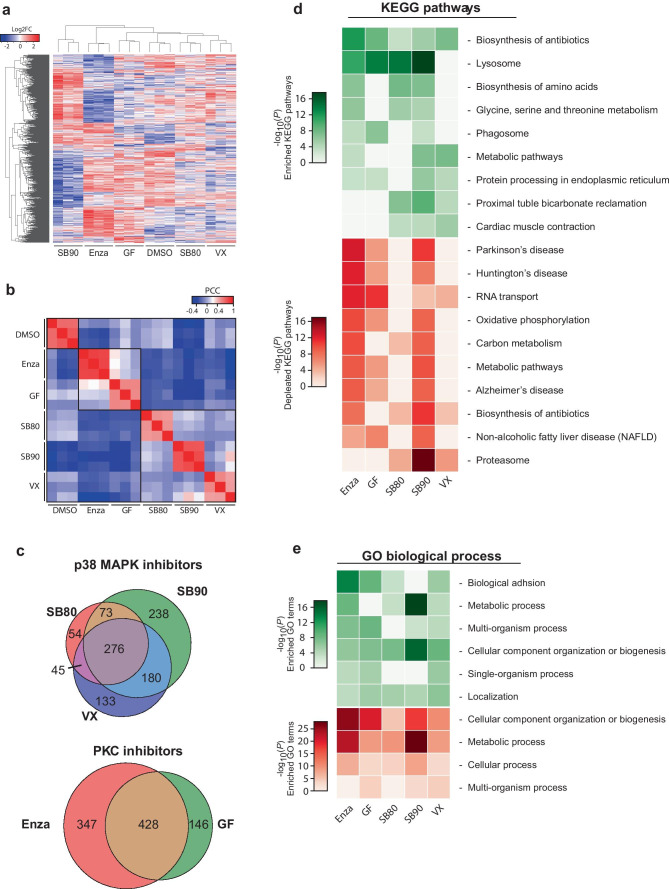
Proteome analyses indicate altered protein levels of lysosome pathways in protective phenotype. **a** Unsupervised clustering of the significantly altered proteome (log2FC) group clusters correctly based on inhibitor and inhibitor target except for SB202190, which is grouped alone. **b** High sample correlation (PCC) within inhibitor treatment confirms reproducibility, while clusters are also observed within inhibitors with the same molecular target. **c** The overlap among the significantly altered proteins of samples treated with inhibitors of the same target is 27.7% and 46.4% for p38 MAPK and PKC, respectively, indicating dissimilar effects of the inhibitors. **d** KEGG pathway analysis reveals downregulation (red) of protein levels within neurodegeneration related KEGG pathways for 3 of 5 compounds. However, lysosome-related pathways show enrichment (green) for 4 of 4 inhibitors with protective phenotype. **e** GO: biological processes remain devoid of alterations matching the observed protective phenotype

From the global proteome, we detected 4989 proteins across all samples, with 1183 proteins being significantly altered when compared to untreated samples. Similar to the phospho-proteome, unsupervised clustering of intensity values from mass spectrometry results in grouped inhibitors based on treatment and inhibitor target (Fig. 5a), with the exception of SB90. SB90 branches off from all other inhibitors, but does however show elevated PCC with SB80 and VX (Fig. 5b). The overlap between the altered proteome for each inhibitor target drops compared to the phospho-proteome, as the proteome only shares 27.7 and 46.4% overlap of significantly altered proteins for p38 MAPK- and PKC-inhibition, respectively (Fig. 5c). This represents a 16.8 and 14.4% decrease in the overlap, respectively.

To identify pathways or molecular events which may be involved in the observed protective phenotype, we performed functional analysis based on the altered proteome. Signatures of particular interest would have to be present for all inhibitors except VX, which did not display the protection from induced α-syn pathology. KEGG pathway analysis, as seen in Fig. 5d, displayed an increase in proteins related to the lysosomal pathway for all compounds with preventative effects on induced α-syn aggregation. This suggests a further investigation of lysosome-related pathways may be warranted.

Of further interest is the decreased abundance of proteins in the pathways mapped in the KEGG database for PD, Alzheimer’s disease, and Huntington’s disease. On further examination of the significantly depleted proteins mapping to these pathways, most were found to relate to mitochondrial-related function (e.g., NDUF, COX and CYC) (see specific log2FC in Supplementary Tables 2–3).

### PKC and p38 MAPK Inhibition Induces Alterations in Endo-lysosomal Compartments

**Fig. 6:**
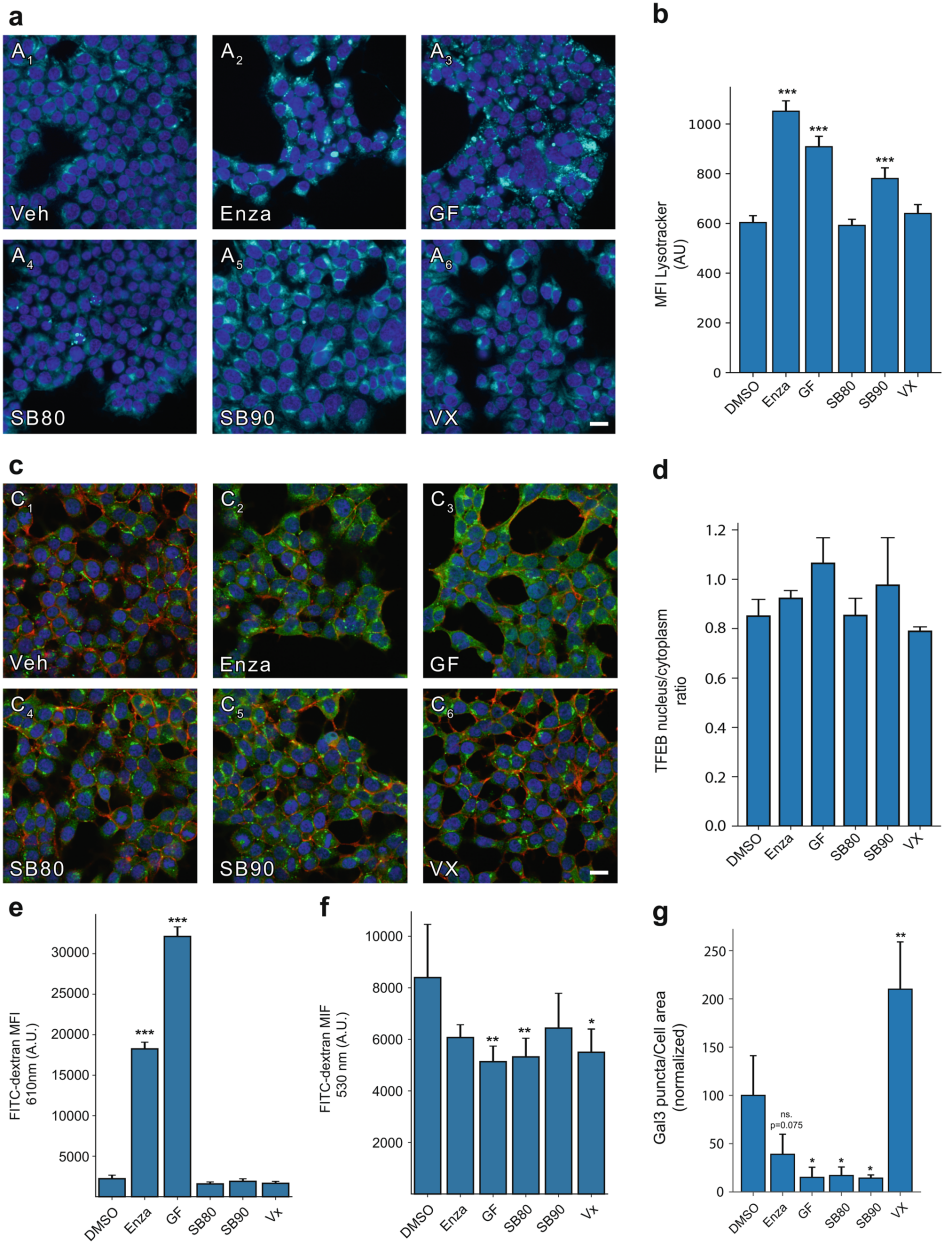
Lysosome and intracellular vesicular alterations in cells treated with hit-compounds. **a** Visualization of acidified compartments by a lysotracker reveals compartments with increased brightness and a more puncta-like appearance (scale 20 µm). **b** the changes in acidified compartments are found to be statistically significant upon flow cytometric analysis for Enza (*p* < 0.001), GF202190 (*p* = 0.001), and SB90 (*p* = 0.001). **c**, **d** Analysis of nuclear translocation at TFEB at 48 h following inhibitor treatment reveal no significant changes in the ratio of nuclear TFEB to cytoplasmic TFEB (scale 20 µm). **e** measuring the total FITC-dextran in the pH-insensitive part of the emission spectrum, we observe a strong significant increase for Enza (*p* < 0.001) and GF (*p* < 0.001). **f** A significant reduction of endocytic uptake was observed by FITC-dextran uptake for GF (*p* = 0.0039), SB80 (*p* = 0.0066), and VX (*p* = 0.0111), with Enza and SB90 trending toward a reduction. **g** Integrity of the endo-lysosomal compartments was examined by galectin3-GFP spot formation 48 h following PFF addition, where spots correspond to permeabilized vesicles. Significant reduction was observed for GF (*p* = 0.0120), SB80 (*p* = 0.0140), and SB90 (*p* = 0.0113), indicating a stabilization of vesicular compartments, and a significant increase for VX (*p* = 0.0019). Bar charts show mean ± SD, *p < 0.05, **p < 0.005, ***p < 0.001. Statistical testing was performed using one-way ANOVA with Dunnett’s T3 post hoc test for multiple comparisons to a control group

To dissect the detailed machinery altered in the endo-lysosomal system as indicated by the altered proteome, we quantified the abundance of acidified compartments. We first visualized acidified compartments by staining inhibitor-treated HEK293T cells with a lysotracker, a compound stain with pH-dependent fluorescence intensity. The abundance of acidified compartments was visualized by live-cell microscopy and quantified by flow cytometry. Significant elevation in acidified compartments was observed for both PKC targeting inhibitors as well as for SB90 targeting p38 MAPK, although to a lesser extent (Fig. 6a, b).

As lysosome-related pathways are in a state of flux, various effects can alter the abundance of lysosome-related compartments, though most commonly this is from lysosomal biogenesis or reduced turnover of lysosomes [29]. We stained inhibitor-treated cells for transcription factor EB (TFEB), the master transcription factor for lysosomal biogenesis, and assessed its activation in the form of nuclear translocation (Fig. 6c). Following inhibitor treatment, no significant increase in the ratio of nuclear to cytoplasmic TFEB was observed (Fig. 6d). The lack of TFEB nuclear translocation indicates that the increase in acidified compartments is not likely to be caused by increased lysosomal biogenesis.

We then sought to investigate the flux of the endo-lysosomal pathway by fluorescein isothiocyanate (FITC) conjugated dextran uptake. While FITC is a commonly applied fluorophore, it is known to be pH sensitive, with 95% of its fluorescence quenched at low pH [30]. The emission spectrum of FITC-dextran can be used to determine the vesicular pH [19]. While pH variation strongly affects the central part of FITCs’ emission peak at 530 nm, where a drop in pH reduces its emission, the tail end of its emission spectrum at 610 nm is less affected by changes in pH [19]. We assessed the uptake of FITC-dextran following a 1-h incubation, at both wavelengths. For the pH-insensitive readout at 610 nm, we measured a statistically significant increase in FITC-dextran only for enzastaurin and GF treated cells, compared to vehicle control (Fig. 6e). However, analyzing the pH-sensitive signal at the 510 nm wavelength, we instead detected a decreased amount of FITC-dextran for GF, SB80, and Vx, while enzastaurin and SB90 exhibit a non-significant decrease (Fig. 6f). This indicates that the increased abundance of FITC-dextran in enzastaurin- and GF-treated cells is localized in low-pH compartments where the fluorescence is quenched.

To investigate the effects on the internalization of α-syn, we employed a cellular model for vesicular permeabilization, using galectin3-GFP (Gal3-GFP). This model develops puncta where vesicles are permeabilized as Gal3-GFP accumulates inside the vesicles. The normalized area of broken vesicles to the total cell area shows a significant reduction in permeabilization events for GF, SB80, and SB90. In line with previous results of aggregate load, VX deviated from other inhibitors by displaying a significant increase in permeabilized vesicles following PFF addition (*p* = 0.0019) (Fig. 6g).

Taken together, the results suggest that PKC and p38 MAPK inhibition can impact seeded α-syn aggregation, likely by preventing vesicular permeabilization and cytosolic entry.

## Discussion

Here, we developed a sensitive and robust cellular reporter for FRET-based detection of α-syn aggregation. Using our cellular reporter, we performed an HTS of small molecule kinase inhibitors, identifying three candidate compounds that diminish seeded α-syn aggregation by inhibiting two actionable therapeutic pathways.

### Development of Cellular α-Syn Aggregation Reporters

When studying protein aggregation, it is essential to select the right model system based on the inherent benefits and limitations. One main factor is the model system’s complexity, from cell-free assays to animal models or patient samples. Cell-free studies have been used to explore different aspects of aggregation, including kinetics and structure [31–33]. Yeast models leverage increased biological relevance while retaining high throughput. Therefore, screening experiments for the study of α-syn and its aggregation have frequently been carried out using yeast models and have identified modifiers of α-syn toxicity, interaction partners, and protein aggregation [34–36].

We sought to establish a model system that would capture important insights into the biology of synucleinopathies. For this purpose, eukaryotic cellular models offered a midpoint between biological relevance and sample throughput. Several cell-based models are available and have been used to study native state interactions [37], such as cell-wide aggregate load and fluorescent fusion constructs to study the cellular aggregation state [38, 39]. However, no model offers direct quantification of aggregates in a workflow easily adaptable to HTS. The primary attributes needed for a model to be fit for HTS is robustness and scalability. We opted to use a FRET-based system as previously reported by Diamond and colleagues for both α-syn and tau [39, 40]. Detection of aggregation through FRET directly measures the aggregation state, which is highly sensitive and can be performed with a flow cytometric workflow for data analyses, as shown in our kinase inhibitor screen.

### Utilizing FRET HTS for Compound Discovery

With advances in automation, HTS can be carried out with thousands of samples, facilitating rapid discovery of potential therapeutic agents and molecular mechanisms. Such screening approaches allow for both unbiased large-scale investigations of mechanisms and targeted screens on specific cellular mechanisms. While larger screens can provide more information and increase the chance of finding targeted events, we opted for a smaller screen as a proof of concept of our model system. Specifically, we selected a small molecule kinase inhibitor library for our screen based on the previous implication of kinases in synucleinopathies [41–45]. Among the 81 compounds screened, we identified 3 potent inhibitors of seeded α-syn aggregation.

While FRET-based detection offers a unique solution for screening for which this monoclonal reporter line has been developed, where it provides a simple experimental setup and analysis workflow, it also has its limitations. Among these is the reliance on both donor and acceptor being present in the same cells in the same ratios and the challenges that arise from dual transduction. The approach we apply here would not be as reliable in primary neurons or animal models, as both parts of the FRET pair are required for functionality. Low viral load risks transduced cells only receiving one FRET component, while high viral load may cause unwanted toxicity. These limitations are however overcome in a screening paradigm as presented here due to the possibility of establishing a monoclonal reporter system. To overcome the limitations related to differential transduction and expression multicistronic vectors or trans-splicing may offer viable solutions. However, attaining accurate stoichiometric control is difficult with the potential exception of 2A self-cleavable peptides [46].

With these limitations in mind, we were able to successfully identify 3 compounds with robust modulation of induced aggregation, clustering on p38 MAPK and PKC, which is supported by previous studies having suggested these pathways as potential therapeutic targets [47–53]. While evidence for a protective role of modulation of these pathways has been suggested, the mechanism by which they might cause the protection is currently not known.

### The Implication of the Endo-lysosomal System

Identifying the molecular changes responsible for the protective effect is difficult when working with broad pathways like p38 MAPK and PKC. Therefore, the use of mass-spectroscopy for an unbiased investigation of the cellular proteome is a valuable methodology for target identification.

The phospho-proteome showed statistically significant alterations in the phosphorylation state of proteins related to protein synthesis, compartment organization, and biogenesis, in line with the inhibitors’ known targets. Both PKC and p38 MAPK are known upstream modulators for a series of molecular functions. PKC is a major regulator for cell division and proliferation [54], while p38 mediates apoptotic/survival gene programs [55], differentiation [56] as well as being involved in inflammation [57].

The proteome instead revealed pathways with potential implications for the observed effect of the inhibitors. A set of mitochondria and metabolism-related proteins mapped to KEGG’s PD, AD, and HD pathways. The involvement of mitochondria and reactive oxygen species for these diseases is well known and documented [58]. However, the downregulation of these KEGG signatures was only present for three of four inhibitors with the protective effects while being absent for SB80 and VX. Instead, an increase in proteins mapped to KEGG’s lysosome pathway was recaptured by all four protective inhibitors. The importance of lysosomal function in relation to synucleinopathies is well supported, with familial variants in glucocerebrosidase (GBA), a lysosomal hydroxylase, being one of the largest risk factors for developing PD [59–61].

When assessing the abundance of acidic compartments, significant increases were only observed for Enza, GF, and SB90, whereas no treatment led to identifiable activation of TFEB driven lysosomal biogenesis. This is contrary to other reports which indicate there is TFEB translocation upon treatment with SB90, corresponding to classical TFEB activation and lysosomal biogenesis [62].

Whether the increased abundance of lysosomes is beneficial or detrimental is still under debate. It is well established that α-syn degradation can be facilitated through chaperone-mediated autophagy, which is lysosome-dependent [59]. However, tau seeding in similar models as ours identifies acidified compartments as a necessary component for tau seeds to enter cells and induce pathology. Disruption of these acidified compartments protected against induced tau aggregation [63].

**Fig. 7:**
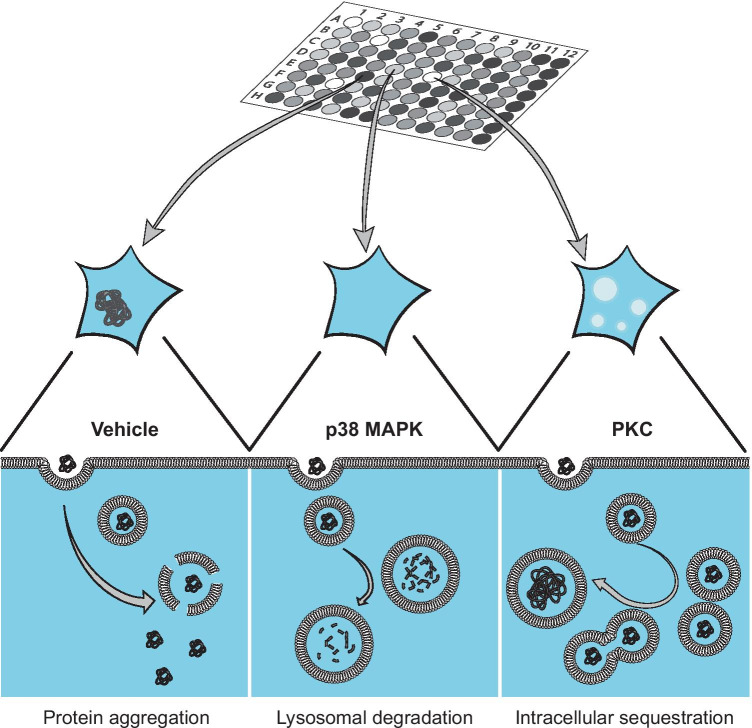
Proposed model for the protective effects of p38 MAPK- and PKC-inhibition on induced α-syn aggregation. The PFF seeded α-syn aggregation paradigm, relies on the extracellular addition of seed material, capable of gaining cellular entry and recruiting endogenous α-syn thereby initiating intracellular aggregation. Upon inhibition of p38 MAPK and PKC by SB80, SB90, and GF, vesicular disruption is prevented. Extracellularly added α-syn may be either degraded or sequestered upon endocytosis, in both cases preventing induction of α-syn aggregation

We further performed a galectin-3 vesicular permeabilization assay and found all 4 protective inhibitors to prevent vesicle permeabilization, with one compound, VX, instead trending towards an exacerbation. The implication of altered vesicle permeabilization would indicate that the inhibitors’ protective effects on induced aggregation occur at or before the point of vesicle permeabilization. This prevention of vesicular disruption could well be caused by increased degradative capacity of lysosomes or sequestration of exogenous detrimental aggregates in vesicular compartments, as indicated by the FITC-dextran uptake for p38 MAPK and PKC, respectively (Fig. 7).

In conclusion, our novel HTS system based on the NPR-H-001 reporter cells provides a new method to study α-syn aggregation and modulation thereof by compounds or other perturbations. Unbiased screening of effectors of α-syn aggregation, as shown here, further allows the identification of molecular mechanisms involved in α-syn pathology. From the screening, we identified three potent inhibitors of induced α-syn aggregation, and through their targets could confirm p38 MAPK and PKC as potential therapeutic targets. Target discovery from screens such as this serves to further deepen our understanding of the molecular mechanisms. In this case, we found further support for the importance of the endo-lysosomal systems in α-syn pathology. The effects observed for the endo-lysosomal system indicated alterations in abundance and degradative capacity was implicated rather than a prevention of internalization of PFFs. Further, more comprehensive screens may serve to broaden our understanding of the molecular mechanisms essential for the induction and maintenance of α-syn pathology.

## Supplementary Information

Below is the link to the electronic supplementary material.

Supplementary file1 (PDF 465 KB)

Supplementary file2 (PDF 272 KB)

Supplementary file3 (PDF 517 KB)

Supplementary file4 (PDF 508 KB)

Supplementary file5 (PDF 416 KB)

Supplementary file6 (PDF 516 KB)

Supplementary file7 (PDF 508 KB)

Supplementary file8 (PDF 495 KB)

Supplementary file9 (PDF 517 KB)

Supplementary file10 (PDF 509 KB)

Supplementary file11 (PDF 463 KB)

Supplementary file12 (PDF 5.04 MB)

Supplementary file13 (PDF 448 KB)

Supplementary file14 (EPS 28 MB)

Supplementary file15 (EPS 6.64 MB)

Supplementary file16 (CSV 3.30 KB)

Supplementary file17 (CSV 452 KB)

Supplementary file18 (CSV 165 KB)
