# Supplementary material for: FRET-Based Screening Identifies p38 MAPK and PKC Inhibition as Targets for Prevention of Seeded α-Synuclein Aggregation
Source: Neurotherapeutics. 2021 Jul 13;18(3):1692–709. doi: 10.1007/s13311-021-01070-1 (PMC8609038; doi:10.1007/s13311-021-01070-1)
Supplement: Supplementary file 8 — Supplementary file8 (PDF 495 KB) [file 13311_2021_1070_MOESM8_ESM.pdf]

Please wait...

If this message is not eventually replaced by the proper contents of the document, your PDF viewer may not be able to display this type of document.

You can upgrade to the latest version of Adobe Reader for Windows®, Mac, or Linux® by visiting http://www.adobe.com/products/acrobat/readstep2.html.

For more assistance with Adobe Reader visit http://www.adobe.com/support/products/acrreader.html.

Windows is either a registered trademark or a trademark of Microsoft Corporation in the United States and/or other countries. Mac is a trademark of Apple Inc., registered in the United States and other countries. Linux is the registered trademark of Linus Torvalds in the U.S. and other countries.

http://www.springer.com/journal/13311

Neurotherapeutics
The Journal of the American Society for Experimental Neurotherapeutics
Editor-in-Chief: Mouradian, M.
ISSN: 1933-7213 (print version)
ISSN: 1878-7479 (electronic version)
Journal no. 13311

Springer

http://www.springer.com/journal/13311

Neurotherapeutics
The Journal of the American Society for Experimental Neurotherapeutics
Editor-in-Chief: Mouradian, M.M.
ISSN: 1933-7213 (print version)
ISSN: 1878-7479 (electronic version)
Journal no. 13311
